# The niche of *Borrelia burgdorferi* sensu lato in Europe is predictable and mappable

**DOI:** 10.1016/j.onehlt.2025.101313

**Published:** 2025-12-24

**Authors:** Agustín Estrada-Peña, Julie Davis, James H. Stark, Patrick H. Kelly

**Affiliations:** a Senior External Consultant, Ministry of Health, Government of Spain, Madrid, Spain; b University of Zaragoza, Zaragoza, Spain; c Global Vaccines Medical Affairs, Pfizer Research & Development, Cambridge, MA, USA; d U.S. Medical Affairs, Vaccines, U.S. Commercial Division, Pfizer, Collegeville, PA, USA

**Keywords:** Europe, *Ixodes ricinus*, Climate, Epidemiology, *Borrelia burgdorferi*, Lyme, One health

## Abstract

Classic environmental niche modelling to examine the distribution of the causative agent of Lyme borreliosis *Borrelia burgdorferi* sensu lato (*Bb*) in the Western Palearctic, is often inadequate because it depends on both its tick vector(s), like *Ixodes ricinus*, and vertebrate reservoirs. We aimed to better determine, identify, and map the geographic distribution of *Bb* genospecies compiling data from 15,032 *I. ricinus* samples and over 6.5 million vertebrate records across 103 genera. We leveraged Species Stacking Distribution Modelling and Principal Components Analysis to identify communities of co-occurring vertebrates and their associations between *Bb* prevalence in host-seeking *Ixodes* nymphs and. Four vertebrate communities were revealed, with one strongly linked as primary reservoirs to *Bb* geographic range. Distribution of *Bb* in southern Europe was limited by the absence of *I. ricinus* despite suitable reservoirs, while in northern regions, a lack of competent reservoirs restricts its spread. The *Bb* prevalence in questing ticks correlates significantly (R^2^ = 0.89) with the presence of key reservoirs rather than overall vertebrate diversity which suggests the *Bb* niche is predictable and tied to specific vertebrate-tick co-occurrences. We compiled a dataset with the climate, vegetation, and vertebrate-derived variables linked to the transmission pressure of *Bb* to humans for the complete European territory, aiming for the prevention of infection in humans. This research underscores the importance of integrating reservoir species and tick distribution data to better map and predict *Bb* spread. By capturing the effects of climate and community composition on the occurrence of *Bb* in Europe, this framework provides insights for tracking Lyme borreliosis at a continental-level.

## Highlights

• *Borrelia burgdorferi* s.l. occupies a niche in the Western Palearctic.
• Climate drives the overlap between *B. burgdorferi* reservoirs and *Ixodes* tick vectors.
• A bioregionalization approach captures occurrence and prevalence in questing ticks.

## 1 Introduction

Tick-borne pathogens (TBPs) represent a significant global public health threat. In Europe some TBPs including the etiological agents of Lyme borreliosis, *Borrelia burgdorferi* sensu lato (*Bb*), are spreading throughout the continent mainly towards greater latitudes and altitudes [1,2]. The distribution and circulation of *Bb* in nature is dependent on the environmental factors that limit the range of its primary vector in Europe, *Ixodes ricinus,* together with multifactorial relationships between a suite of abiotic (e.g. climate and land use) and biotic (e.g. hosts for ticks and reservoirs for the pathogen) factors that influence local infection and transmission dynamics [3].

It is well documented that local vertebrate community composition and richness (β-diversity) strongly influence the circulation and amplification of TBPs in natural foci, including the prevalence of *Bb*-infected ticks and epidemiology of Lyme borreliosis [[4], [5], [6]]. The high diversity of vertebrate communities in Europe provides variable combinations of mammals, birds, and lizards serving as tick hosts and *Bb* reservoirs. Notably, individual *Bb* genospecies possess varying preferential reservoir specificity and pathogenic capacity [7]. For instance, *Borrelia burgdorferi* sensu stricto has broad geographic niches in both Europe and North America and utilizes many reservoir species while (e.g.) *Borrelia spielmanii* or *Borrelia lusitaniae* have narrower reservoir host ranges within Europe exclusively [8,9]. These phylogenetic associations are presumed to be an indicating hallmark effect of historical speciation events [10]. Therefore, estimating the geographic ranges of *I. ricinus* and *Bb* should consider their associations with relevant environmental variables to more reliably identify the factors that most affect their distributions.

We previously demonstrated that *Bb* distribution follows a climate niche in the Western Palearctic, associated to unique vertebrate community compositions [[11], [12], [13]]. These studies led to the development of novel ecological concepts regarding the “bioregionalization” of both vertebrates and their ticks across large-scale geographies, which lead to identifying areas with similar epidemiological characteristics that influence TBPs distribution. However, a proof-of-concept unifying the current knowledge on the *Bb*-reservoir-*Ixodes* associations and their abiotic factors is lacking. Harmonizing the spatial occurrence and abundance data for individual vertebrate reservoirs and hosts for *Bb* and *Ixodes* ticks with other relevant explanatory variables into a single model would provide valuable information to the specific geographic niche(s) for each *Bb* genospecies.

Our study intends to establish that the geographic ranges of *Bb* result from unique compositions between *I. ricinus* and vertebrate communities and show how specific ecological niches for *Bb* result from the co-occurrence of key *Bb* reservoirs with *Ixodes* vector, driven by climate. We also aimed to interpret how the interaction and co-occurrence between vertebrate communities and *I. ricinus* provide an ecological niche to model the prevalence of *Bb* infection in questing nymphs across the continent. We projected the model into a geographical analysis of Europe, aimed to pinpoint the “hot spots” in the continent according to a multi-criteria decision analysis based on the presence of the *Ixodes* tick vector, predicted prevalence of *Bb*, vertebrate community composition, landscape and forest structure, populated places and human population. To our knowledge, this study incorporates the most comprehensive vertebrate reservoirs and tick hosts critical for *Bb* circulation in Europe and provides the first Europe-wide model mapping the unique ecological niches for each *Bb* genospecies illustrating important epidemiological patterns of Lyme borreliosis risk in humans.

## 2 Material and methods

### 2.1 Retrieval of vertebrate, tick, and climate data

Vertebrate, tick, and climatic data for modelling were extracted across a target region from 26°W, 71°N (upper left corner) to 57°E, 30°N (lower right corner). For vertebrate distributions, we utilized a previously compiled dataset [4] that includes vertebrate species reported as tick hosts over the past 40 years [11,[14], [15], [16]] updated with (non-duplicative) records from the Global Biodiversity Information Facility (GBIF) [17] (full list provided in Supplemental File S1). Records were processed at the genus level to optimize geospatial harmonization across millions of occurrences. In total, the dataset contained 6,538,201 vertebrate occurrences across 103 genera. Records for *I. ricinus* were sourced from five previous compilations [4,11,[18], [19], [20]] including details on their georeferenced occurrences. After removal of redundancies, the final dataset included 15,032 unique observations of *I. ricinus* restricted to the Western Palearctic.

Climate data were extracted from the TerraClimate repository at a spatial resolution of 4 km [21]. Data on the maximum temperature (TMAX), minimum temperature (TMIN), and water vapor pressure deficit (VPD) were compiled and averaged at monthly intervals between 1990 and 2020 using the “terra” package [22] in R. We performed harmonic regression on the 12 averaged monthly values (temperature or water vapor deficit) against time (from months 1–12). The coefficients derived from harmonic regression were used as the explanatory variables for tick or vertebrate occurrence modelling. These variables are orthogonal and non-self-correlated, eliminating the need to assess collinearity among variables for good modelling practices. They also retain ecological relevance for environmental suitability modelling and capture critical conditions for organism survival better than sets of pre-tailored climate data [23]. Nine variables were used to describe climate, namely the first three coefficients of the harmonic regression for TMAX, TMIN, VPD.

### 2.2 Model development and approaches

**Fig. 1:**
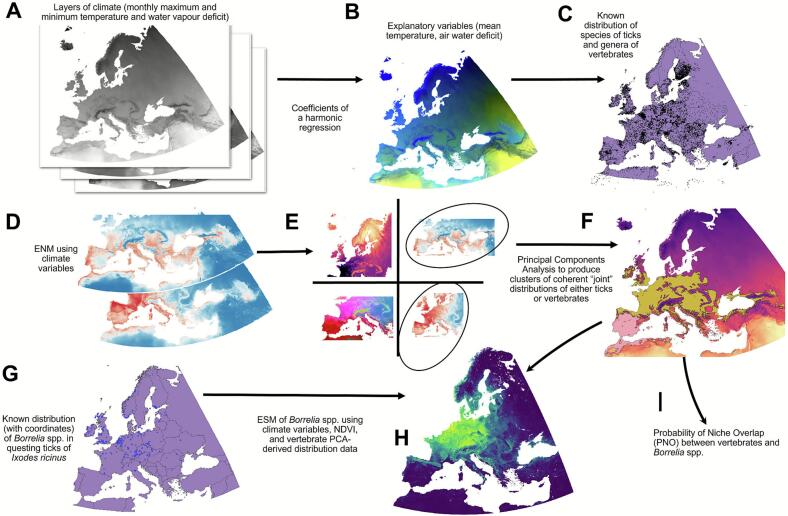
Schematic flow diagram of model study. Climate data layers of temperature and water vapor deficit (A) were used to model the distribution of 11 species of ticks and 86 genera of vertebrates in the western Palearctic. A harmonic regression was fitted to the average monthly values of maximum and minimum temperature, and water vapor deficit; the coefficients of the regression were used as explanatory variables (B). Reported and/or recorded distribution of ticks and vertebrates was obtained from different sources (C) and used to train models for each tick or vertebrate (D) resulting in environmental niche modelling of each taxon. The distribution of the 86 genera of vertebrates was subjected to Principal Components Analysis (E) to detect overlapping distributions as clusters of co-occurring vertebrate genera, also known as chorotypes (F). The reported records of *Borrelia burgdorferi* s.l. in questing *Ixodes ricinus* nymphal ticks (G) were used to map the expected distribution of the bacterium in questing ticks by a multiple regression, using exclusively the range of *Ixodes ricinus* and key vertebrates detected by the PCA.

We used a species stacked distribution modelling (SSDM) approach leveraging simultaneous ensemble modelling with multiple algorithms to select the best performing model (See Fig. 1) [24]. An ensemble modelling approach enables the capturing of potential interactions among vertebrates and their relationships with climate variables, yielding more accurate representations of their actual distributions [25]. We used the SSDM package in R [26] to predict the expected distributions of *I. ricinus* and vertebrates, separately, using the best ensemble models using the GAM, MARS, and SVM algorithms. For GAM, we ran 500 iterations per species/genus with an epsilon value of 1E^−8^; for MARS, we allowed up to 4 degrees of interactions; for SVM, we used an epsilon of 1E^−8^ with 3 cross-validation steps. Unspecified parameters were retained at their default SSDM values. Model training and testing were performed at a 70:30 (training:testing) of the available data. Pseudo-absences across the target territory were generated automatically by the software matching the organism records 1:1. Vertebrate ß-diversity and endemicity were measured as described [27,28]. We used the corrected weighted endemism index (CWEI) to calculate the inverse sum of the geographical range for each species at the desired spatial scales. Model performance and selection for ensemble SSDM were based on specificity (proportion of correctly predicted absences) and sensitivity (the proportion of correctly predicted presences) threshold values, the omission rate (proportion of missing records with the best model), accuracy (proportion of correctly detected records), and Cohen's kappa. Models were compared via area under the curve (AUC) but its use was restricted to comparisons within the same species.

### 2.3 Clustering procedures

The range maps of the tick and vertebrates were used to (1) establish *Bb-Ixodes-*vertebrate relationships; and (2) calculate vertebrate chorotype clusters as published previously [29]. A chorotype is a set of species that co-occur in a significant way resulting in a common range. Chorotypes are the roots of an epidemiological regionalization because they consist of the identification of spatial units with similar species composition.

We applied a clustering procedure based on Principal Components Analysis (PCA) across the 103 vertebrate genera. The aim of PCA was both to reduce the spatial variability into axes that explained model variance and to define spatial epidemiological units (variable chorotypes) to illustrate *Bb* distributions. Clusters were created using the k-means algorithm, to optimize the number of clusters required to explain observed variability through the “NbClust” [30] and “factoextra” [31] packages using the Gap Statistics method. The proximity of each vertebrate species to the PCA axes indicated its membership to a chorotype of vertebrates (hereby referred as “clusters”) sharing similar ranges.

### 2.4 Variable associations and modelling Bb distribution and prevalence in questing nymphs

We updated previously compiled datasets of georeferenced prevalence records for *Bb* in questing *I. ricinus* nymphs [18,20]. The nymphal tick life stage was selected because it has been already infected while feeding as larva and is free of contamination from the blood of the hosts if collected while feeding. The predicted distribution of *Bb* infected *I. ricinus* was calculated using descriptive variables for endemicity and β-diversity of vertebrates, the range of vertebrate clusters, *I. ricinus* distribution, and the climate variables. The best model for *Bb* distribution within questing *Ixodes* nymphs was obtained using the same procedures as for the other models. To determine which vertebrate cluster(s) best described the distribution of *Bb* infected *I. ricinus* nymphs in the model, we measured their spatial co-occurrences by comparing the pixel-by-pixel habitat suitability via predicted niche overlap (PNO) using the R package “phyloclim” [32] and through Schoener's D distance [33] using a previously described method overlaying a 18,861 hexagonal cell grid on the vertebrate cluster distributions across the entire geographic territory [12,19]. Each hexagonal cell contains the median suitability value for each vertebrate species within the territory (10 km radius) in addition to the prevalence of *Bb* in questing nymphs. The variables for vertebrate cluster distributions that best explained *Bb* distribution and prevalence in questing nymphs were identified according to multiple regression analysis and variable importance was measured by Pearson's correlation score 1 – r between the final modeled variables and iterative variables omitted to measure each variables' model contributions.

### 2.5 A dataset for decision making and prevention of Bb in humans

Lastly, we sought to incorporate the known environmental determinants of *I. ricinus* and *Bb* distribution into the hexagonal cell grid model to illustrate important epidemiological determinants of Lyme borreliosis that informs human-related risks in Europe. The synthesized dataset is meant to define the ecological attributes that identify high-risk areas with increased likelihood of *Bb* exposure and infection in humans. Similarly, we utilized the hexagonal grid to integrate the additional variables for human population and features of landscape with the explanatory variables for climate, vertebrate species, and *I. ricinus* nymph and *Bb* occurrence into the associated 18,861 cells. Data for the landscape variables (dominant vegetation type, habitat fragmentation, and proportion of forest type and forest cover) were extracted from the CORINE Land Cover 2018 inventory at 100 m resolution (available at https://land.copernicus.eu/en/products/corine-land-cover) and public data repositories from Copernicus as detailed. We used human population data from the SEDAC Gridded Population of the World (available at https://earth.gov/ghgcenter/data-catalog/sedac-popdensity-yeargrid5yr-v4.11), selecting the grid for the year 2020.

## 3 Results

### 3.1 Vertebrates and tick distributions follow climatic gradients

**Fig. 2:**
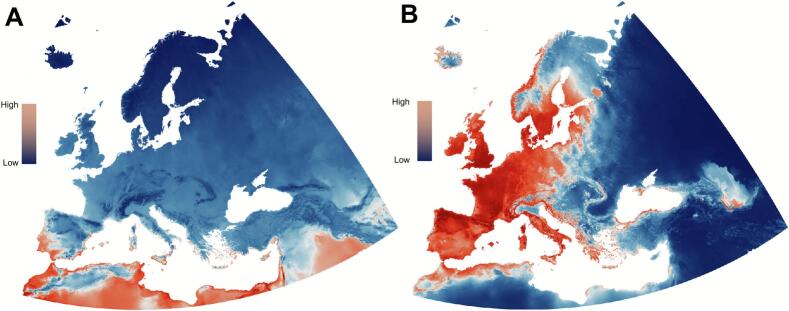
Modeled spatial suitability of the vertebrate genera and *Ixodes ricinus* across the target territory. Predicted vertebrate genera endemicity (A), ß-diversity (B) and predicted distribution of *Ixodes ricinus* (C) is shown at a scale of 0–100.

**Fig. 3:**
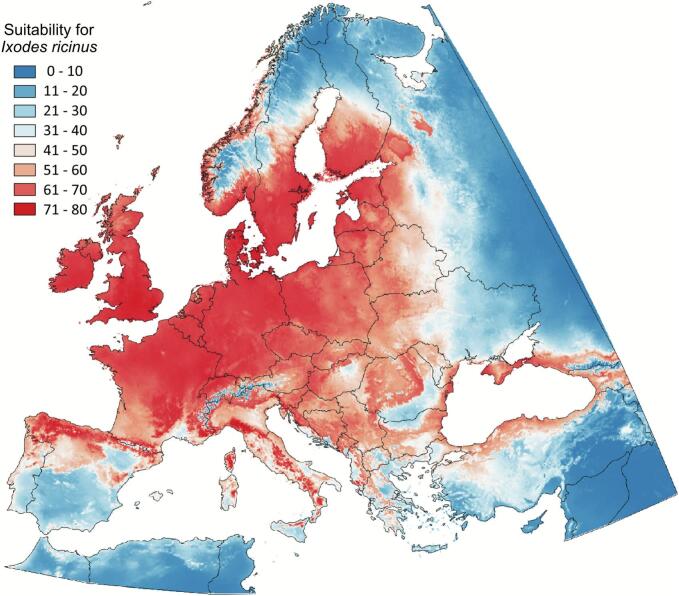
The predicted distribution of *Ixodes ricinus* nymphs (C) in the target territory on a scale of 0–100.

Model performance for the 103 vertebrate genera is provided in Supplementary File S2. The best models to predict vertebrate genera distributions yielded an average AUC of 0.93, with a sensitivity and specificity of 76 % and 86 %, respectively. Areas with the highest vertebrate endemism were confined to the southwestern Iberian Peninsula and regions in northern Africa (Fig. 2A). High vertebrate β-diversity spanned large areas in western Europe, with the highest observations in northern Iberia, western France, and southern United Kingdom (Fig. 2B). The best model for *I. ricinus* distribution yielded an average AUC of 0.96 with an 89 % sensitivity and 88 % specificity. The highest predicted suitable areas for *I. ricinus* spanned from the northern Iberian Peninsula across Central Europe (France, Germany, and Poland) to the Baltic states (Latvia and Estonia) and northward throughout the United Kingdom into the southern Nordics of Finland, Sweden, and Norway (Fig. 3). Areas with moderate predicted suitability for *I. ricinus* extended into northern and higher altitudes of Italy, Greece, through the Balkans-Carpathian plateau into Greece and northern Turkey, reaching eastern Europe in Belarus and Ukraine (Fig. 3).

### 3.2 Vertebrates have co-occurring ranges in western Palearctic

**Fig. 4:**
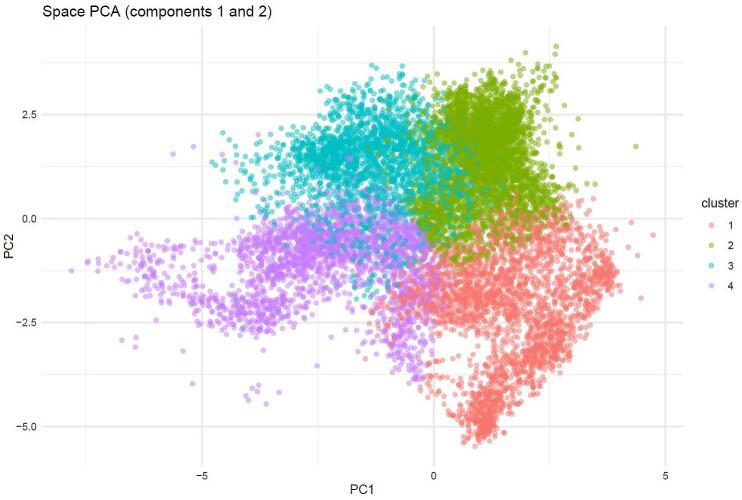
Principal Components Analysis (PCA) carried out on the distribution of the 103 vertebrate genera included for analysis. Each dot corresponds to one hexagon of the hexagonal coverage over the target territory. The position of each dot represents individual vertebrate genera associations with the principal axes 1 and 2. The chart shows four statistically distinct clusters of co-occurring vertebrate genera (with separate geographic distributions.

**Fig. 5:**
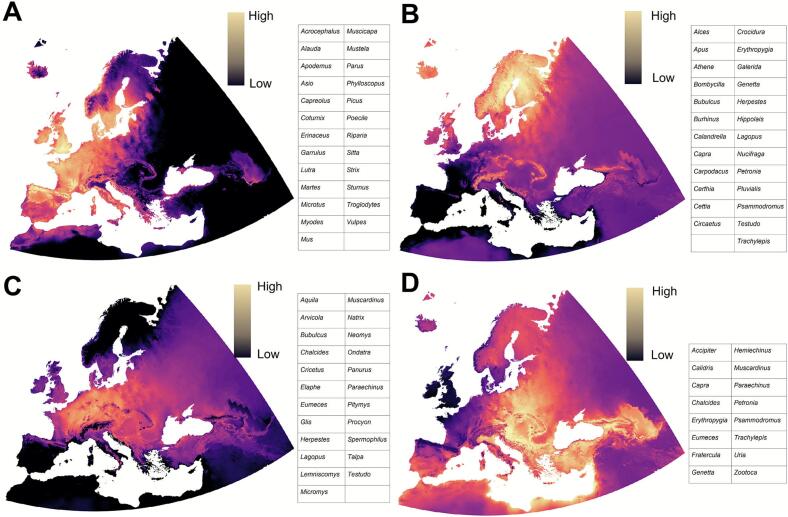
The geographic distribution of co-occurring vertebrate clusters in the studied area. Each figure corresponds to the vertebrate genera with overlapping territories defined as Cluster 1 (A), Cluster 2 (B), Cluster 3 (C), and Cluster 4 (D). Colors indicate high or low niche suitability for the vertebrate clusters.

The predicted distributions of *I. ricinus* ticks and vertebrate hosts/reservoirs were used to identify unique vertebrate community “clusters” across the continent and indicate where spatial foci of *Bb* genospecies are most probable. These clusters capture statistically significant vertebrate co-occurrences and define unique spatial patterns in their geographic distributions across Europe (Fig. 4). Four vertebrate clusters were identified, with the strongest PCA contributions observed in clusters one (59 %) and two (41 %) (Fig. 4). Cluster 1 (Fig. 5A) included 21 vertebrate genera that most strongly co-occurred throughout Central Europe and the United Kingdom, extending northward along coastal Norway and southern Sweden to the southern Iberian Peninsula, primarily in European areas with low climatic continentality. Cluster 2 (Fig. 5B) describes the ranges of 25 vertebrate generate occurring in European mountain ranges and colder regions, spanning from the Nordic countries into the northern United Kingdom. Cluster 3 (Fig. 5C) included 23 vertebrate genera restricted to continental and eastern Europe, avoiding mountainous European regions of southern France and Switzerland, and diffusing across eastern Europe. Cluster 4 (Fig. 5D) includes 16 vertebrate genera co-occurring most strongly across eastern Europe in Belarus, Ukraine, and the Balkans. This cluster is less defined and appears to represent residual distributions that could not be statistically assigned to other clusters.

### 3.3 *Borrelia burgdorferi* sensu lato genospecies possess distinct ecological niches

**Fig. 6:**
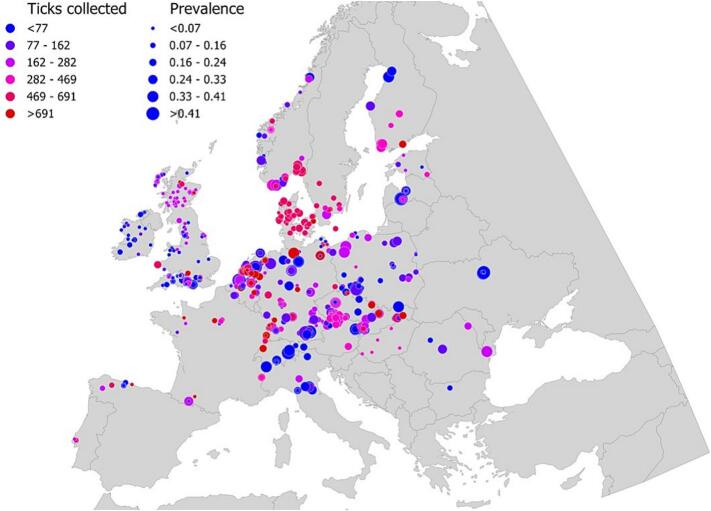
Recorded distribution and prevalence of *Borrelia burgdorferi* sensu lato in the target region in Europe, as published (summarized in [22]). Only georeferenced records reported for questing nymphs of *Ixodes ricinus* are included. Colors represent the number of nymphal ticks examined. The size of each dot is proportional to the prevalence of *Borrelia burgdorferi* sensu lato in the examined ticks.

**Fig. 7:**
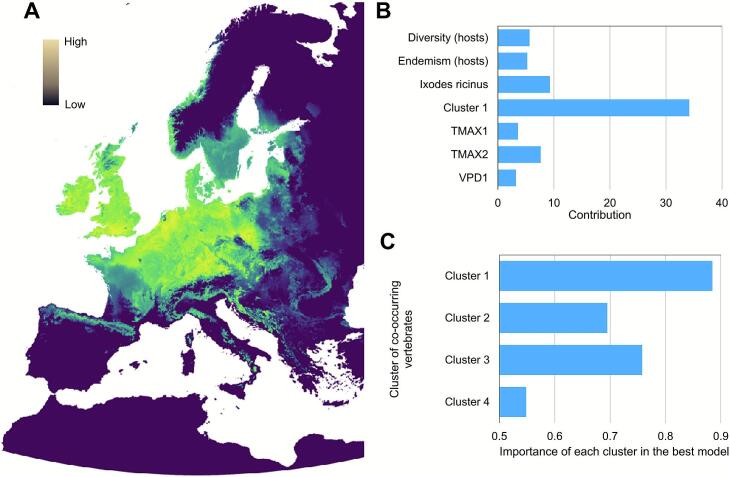
The predicted geographic distribution of *Borrelia burgdorferi* sensu lato in the target territory. (A) modeled according to explanatory variables associated with *Ixodes ricinus,* climate, landscape, and vertebrate species. Histogram of the contribution of the seven variables that built the best model to predict the distribution of *Borrelia burgdorferi* in *I. ricinus* (B). The contribution of each vertebrate cluster in the best model to predict the distribution of *Borrelia burgdorferi*, in a range of 0–1 (C); the vertebrate cluster that best defines the expected geographic range of *Borrelia burgdorferi* is Cluster 1.

**Fig. 8:**
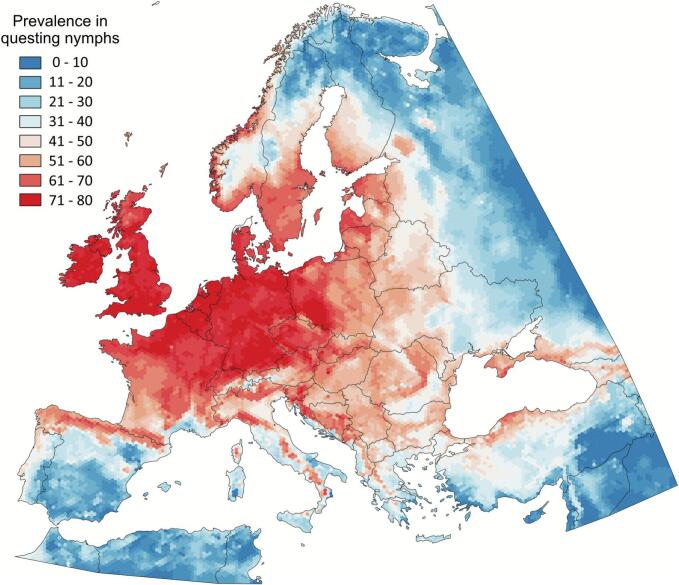
The predicted prevalence of *Borrelia burgdorferi* sensu lato in questing *Ixodes ricinus* nymphs in the target territory.Values (%) of *B. burgdorferi* prevalence are calculated via a hexagonal grid system consisting of 18,861 cells overlying the study territory, incorporating the suitability data of 21 vertebrate genera from Cluster 1.

Collectively, 154 records yielded the extraction of 276,440 *I. ricinus* host-seeking nymphs tested for *Bb* infection yielding a total of 2370 unique georeferenced observations (Fig. 6). The Random Forest algorithm was the best performing model to predict the spatial distribution of *Bb*, yielding an AUC of 0.99 with 94.4 % sensitivity and 94.9 % specificity. The expected probability for the predicted distribution of *Bb* in questing *I. ricinus* nymphs is illustrated in Fig. 7A. The variable for the distribution of Cluster 1 vertebrates was the strongest contributor to the overall model for *Bb* geographic range, followed by variables for *I. ricinus* distribution, vertebrate β-diversity, vertebrate endemism, and three climate-derived variables including the spring-slope temperature (Fig. 7B). To note, high PNO values of the predicted *Bb* distribution relative to Cluster 1 demonstrate the strong spatial correlation between *Bb* and the respective 21 vertebrate genera (Fig. 7C). Table 1 includes the results of a multiple regression (R^2^ = 0.859) between the prevalence of *Bb* in *I. ricinus* nymphs and Cluster 1 vertebrates. The details of the multiple regression analysis are provided in Table 2. All independent variables for each vertebrate genus were highly significant (*p* < 0.0001) in the model, except for *Apodemus* (*p* = 0.0755). Multiple regression analyses using the distributions of vertebrate Clusters 2–4 had reduced explanatory variance (R^2^ < 0.6) demonstrating Cluster 1 with the strongest explanatory power to describe the range and prevalence of *Bb*. Using the aggregated outputs for entire 18,861 hexagonal cell grid, the predicted prevalence of *Bb* in questing *I. ricinus* nymphs was calculated and illustrated in Fig. 8.

**Table 1:** Results of a multiple regression of the prevalence of *Borrelia burgdorferi* sensu lato (dependent variable) using the distribution of 21 vertebrate genera that conform the Cluster 1. Other than the value of the squared regression, a summary of the analysis of variance is included.

Source	Degrees of Freedom	Sum of squares	Mean square	F-Ratio	*P*-Value
Intercept	1	1465.005	1465.005		
Model	21	1517.464	72.26018	5457	0.0000
Error	18,833	249.378	0.013241		
Total (Adjusted)	18,854	1766.842	0.09371

**Table 2:** Details of the analysis of variance of the multiple regression of the prevalence of *B. burgdorferi* s.l. in the target area in Western Palearctic. Included are the 21 vertebrate genera (Cluster 1) used as independent variables for the multiple regression (column “source”). Other columns include the R^2^ of the model if the vertebrate genus is removed, as well as the sum of squares, the mean square, F-Ratio and P-Value.

Source	Degrees of Freedom	R^2^ Lost if removed	Sum of Squares	F- Ratio	P-Value
Intercept	1		1465.005		
Model	21	0.8589	1517.464	5457.068	0.0000
*Acrocephalus*	1	0.0001	0.2478359	18.716	0.0000
*Alauda*	1	0.0092	16.25772	1227.778	0.0000
*Apodemus*	1	0.0000	0.04182822	3.159	0.0755
*Capreolus*	1	0.0174	30.70046	2318.490	0.0000
*Coturnix*	1	0.0002	0.3123466	23.588	0.0000
*Erinaceus*	1	0.0013	2.255273	170.318	0.0000
*Garrulus*	1	0.0007	1.1581	87.459	0.0000
*Lutra*	1	0.0161	28.42175	2146.402	0.0000
*Martes*	1	0.0021	3.731594	281.809	0.0000
*Microtus*	1	0.0003	0.5026489	37.960	0.0000
*Mus*	1	0.0001	0.2629999	19.862	0.0000
*Myodes*	1	0.0237	41.84777	3160.332	0.0000
*Muscicapa*	1	0.0006	1.10444	83.407	0.0000
*Mustela*	1	0.0005	0.9492995	71.691	0.0000
*Parus*	1	0.0007	1.303021	98.404	0.0000
*Phylloscop*	1	0.0004	0.660497	49.881	0.0000
*Poecile*	1	0.0084	14.91253	1126.190	0.0000
*Strix*	1	0.0002	0.3307413	24.977	0.0000
*Sturnus*	1	0.0062	11.01786	832.065	0.0000
*Troglodytes*	1	0.0003	0.4426688	33.430	0.0000
*Vulpes*	1	0.0003	0.5741248	43.358	0.0000
Error	18,833	0.1411	249.3786		
Total (Adjusted)	18,854		1766.842

### 3.4 A dataset for decision making and prevention of Bb in Europe

Since our models demonstrated high accuracy to predict the ecological niche of *Bb* in Europe, we next sought to incorporate additional epidemiological variables relevant for *Bb* transmission of in humans to build a publicly available spatial dataset exploring potential “hotspots” of Lyme borreliosis. Ultimately, the aim for constructing this spatial dataset package is two-fold: 1) examine the probability of *Bb* transmission across Europe and 2) define probable regions where public health protection measures would be useful to prevent human exposure of *Bb-*infected *I. ricinus* nymphs.

**Fig. 9:**
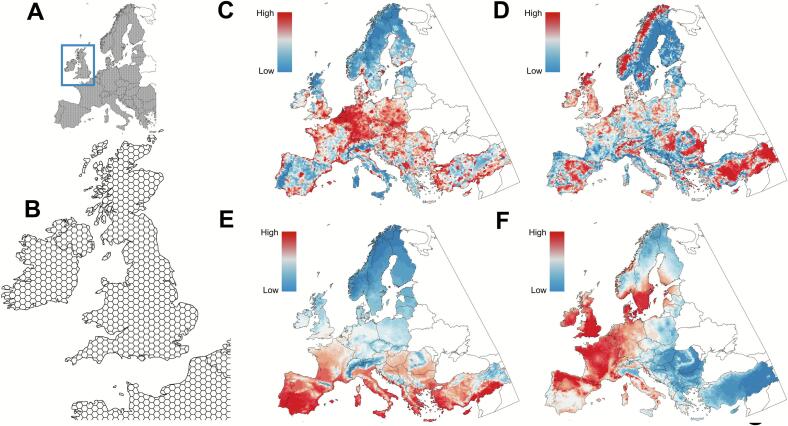
Spatial output of the comprehensive GeoPackage dataset for the study to model enzootic risk of Lyme borreliosis in Europe. Dataset includes all explanatory variables for *Bb* distribution and prevalence in Europe in the study. The dataset and associated variable data are provided in Supplemental File S3 and available for public download via GeoPackage. A schematic of the spatial resolution of the hexagonal dataset (A-B). Featured outputs from the package demonstrating spatial correlations with *Bb* infection and enzootic risk of Lyme borreliosis are shown for human population (C), magnitude of forest fragmentation (D), mean annual temperature (E), and abundance of reservoirs (F).

Collectively, the dataset aggregates the observed explanatory variable data respective to each 10 km^2^ hexagon across the entire grid system in a comprehensive package incorporating the i) landscape features for habitat composition, forest fragmentation, forest type, and forest canopy distributions; ii) the predicted distributions of *I. ricinus* nymphs and prevalence of *Bb*; iii) vertebrate β-diversity and suitability of vertebrate *Bb* reservoirs; iv) climatic variables for temperature (TMAX, TMIN) and VPD; and v) human population. The dataset is provided as Supplemental File S3 and made available as a GeoPackage, a file format compatible with most open access geographical information systems (GIS) software and importation with other statistics packages for mapping or additional modelling procedures. The spatial resolution of the hexagonal dataset is sketched in Fig. 9A-B. Featured outputs from the dataset package to correlate *Bb* infection and enzootic risk of Lyme borreliosis are provided for human population (Fig. 9C), magnitude of forest fragmentation (Fig. 9D), mean annual temperature (Fig. 9E) or abundance of reservoirs (Fig. 9F).

## 4 Discussion

Climate and landscape features drive the patterns of co-occurrence of ticks and vertebrates that shape the ecological communities in which tick-borne pathogens circulate. To date, the distribution and prevalence of *Bb* has not been reliably predicted and mapped in Europe, likely due to limited sampling of highly influential explanatory variables (e.g., vertebrates), high spatial aggregation / correlation of available records that precludes successful identification of important explanatory variable associations, or the need for a higher-resolution analysis. This was a fundamental gap in public health, limiting public awareness of when and where personal protection measures should be adopted to mitigate the risk of tick-borne diseases. We aimed to address this gap by mapping the distribution of *I. ricinus* and prevalence of *Bb* representing their expected ranges in the Western Palearctic using a novel predictive approach incorporating vertebrate endemicity, β-diversity and abundance of vertebrate host and *Bb* reservoir genera. To our knowledge, this study is the first to identify a linked spatial correlation over such a large territory between clusters of specific co-occurring vertebrate genera and *Bb* occurrence and prevalence in questing *I. ricinus* nymphs. This is the best effort to date to predict the distribution of established *I. ricinus* populations and inform local enzootic risk of *Bb* transmission to prevent infective tick bites to humans.

Our study demonstrated that the community composition of unique vertebrate clusters greatly influences prevalence and distribution of *Bb* infection in questing *I. ricinus* nymphs, as hypothesized from other field studies [34,35]. It is well known that certain vertebrate species are highly competent reservoirs that amplify *Bb* in the blood to most efficiently infect feeding ticks [7,36]. Other vertebrates are incompetent *Bb* reservoirs but serve as tick maintenance hosts for bloodmeal acquisition [37]. Vertebrate ß-diversity and abundance, thus, significantly impact *I. ricinus* occurrence and population dynamics, and subsequent *Bb* circulation based on the homeostasis between specific vertebrate hosts that drive tick abundances (e.g., deer, boar) and competent reservoir species that increase *Bb* infection in ticks (e.g., rodents, insectivorous, small birds). In the context of our study, 20 of the 21 vertebrate genera in Cluster 1 represent the most critically important tick hosts and *Bb* reservoirs influencing the distributions of *I. ricinus* and *Bb* prevalence than any other vertebrate community clusters included for analysis.

We demonstrate that *Bb* has a predictable niche in Europe with distinct climatic features. Climate defines the distribution of *Ixodes* vectors and key host species and vertebrate β-diversity for blood feeding and life cycle development. Furthermore, *Bb* prevalence in questing *I. ricinus* nymphs can be mapped with high reliability using the relative abundance of vertebrates that co-occur across large regions. The modeled niche outputs are critical to provide better insights into the future geographic range and habitat suitability of *Bb* and *Ixodes* ticks in Europe due to climate change, which is projected to result in the increased burden of tick-borne diseases across additional regions [38]. This would reshape the epidemiological patterns of Lyme borreliosis in Europe based on the climate features that directly impact the vertebrate community clusters that are strongly associated with *Bb* circulation as demonstrated in this study.

Classically, predicting the geographic range of tick-borne pathogens has utilized many approaches using inconsistent or incomplete sets of climate- and landscape-derived variables [39]. Despite the well-known role of vertebrate hosts and *Bb* reservoirs, few large-scale models have included co-occurring animal communities as data inputs. Results of this study point to several vertebrate species that co-occur across a large territory and statistically cluster within distinct geographies of their known occurrences which strongly predict *Bb* occurrence. This is interpreted as the distributions of certain vertebrate reservoirs [40,41] and *I. ricinus* overlap where human cases of Lyme borreliosis in Europe are most common.

Importantly, this study is restricted to the *Bb* and *I. ricinus* system. It is known that *Bb* expands in the Palearctic further east where *Ixodes persulcatus* replaces *I. ricinus* due to its greater tolerance for colder temperatures [42]. Our estimation of the faunal composition and its impact on the occurrence and prevalence of *Bb* is a metric akin to density of infected nymphs (DIN) considered the best metric to inform the likelihood of a tick encounter and human risk of Lyme borreliosis [43]. Clusters extending into Northern Africa where *Bb* can occur though local populations of *I. ricinus* may not be well established [44]. Although some reliably geo-referenced records of questing *I. ricinus* nymphs in the southern fringe of its range have been identified [45] the zone was unable to be included for model training. Therefore, our maps probably underrepresent the expected distribution of *Bb* due to the lack of geo-referenced records in under-surveyed regions. Despite these limitations, the predicted distributions clearly summarize the key concept of the study: the co-occurrence of distinct vertebrate genera is essential for *Bb* occurrence and can be mapped based on climate and their community composition which directly impacts *Bb* transmission risk to humans.

A critical goal of this study was to develop a predictive model that allowed us to produce the largest publicly available and most comprehensive explanatory variable dataset in terms of extension and biotic and abiotic variables to measure Lyme borreliosis risks in Europe. These explanatory variables include the distribution of the reservoirs, something that was not previously available, as one of the main biotic factors outlining the range and prevalence of *Bb*. We sought to achieve this goal by packaging the dataset with the most relevant epidemiological variables influencing *Bb* transmission to humans. Importantly, data for biotic variables of vertebrate hosts and reservoirs and humans are notably available only for local or regional modelling studies for tick-borne diseases [[5], [6], [7],^40^,^46^]. As such, the dataset provided from this study is robust and can strongly augment other statistical analyses and/or modelling studies evaluating the risk of Lyme borreliosis (and individual variables driving it) in Europe. Ultimately, the data package can serve as a basic tool to improve human health and prevention of tick-borne diseases.

Nevertheless, this study has some gaps and limitations. Mapping the range of vertebrates across a large Western Palearctic territory with diverse ecosystems and environmental traits may lead to the non-inclusion of vertebrates from the eastern edge of their distributions and indicate a lack of suitable habitats in eastern territories. Given the models high-performances, additional records of *I. persulcatus* and their hosts could be applied for broader spatial predictions geographies (e.g. western Russia) without sacrificing reliability. Another drawback modelling *Bb* prevalence in questing nymphs is the lack of zero prevalence values (e.g. negative data) which typically go unreported. There are many non-surveyed hexagons in the honeycomb overlying the selected territory which we accounted for this by incorporating pseudo-absences observations during model training to reduce overfitting, though unlivable areas of *I. ricinus* in high altitudes (>2000 m above sea level) [47] and northern latitudes (<66 N) [48] could be applied. We acknowledge that the zero-prevalence observations could increase bias, but regressions based on zero-inflated negative distribution provided poorer performance metrics and the method was rejected [46]. The modelling framework in this study focused on vertebrate compositions and climate to evaluate the spatial variability of *Bb* across large areas at coarse resolution. The high-resolution effects of local landscapes on this system could likely be addressed with the incorporation of additional abiotic variables identified in other regional models trained with field studies [49,50]. Such an effect of landscape features on host composition may have a local nature, since changes of *Bb* prevalence in questing nymphs have been repeatedly demonstrated under many conditions and reservoirs community composition.

## 5 Conclusions

Climate shapes the distributions of TBPs, through indirect impacts on vectors, hosts, and reservoirs. Future range changes of key vertebrates (driven by climate) could modify the limits of biotic barriers, or yet unpredicted host shifts, that could be key drivers of TBPs retreats or invasions. The framework model in this study could track the impact of climate change on *Bb* circulation, since it includes explanatory variables with ecological relevance, the spread of co-occurring key vertebrates, and environmental suitability for the main tick vector in the studied territory. It is also a suitable method for understanding the underlying epidemiological pathways of TBPs with varying interactions across climate, ticks, and vertebrates, which are not yet well inferred.

## Availability of data and material

All the raw data used for this study are available as Supplemental Material.

## CRediT authorship contribution statement

**Agustín Estrada-Peña:** Writing – review & editing, Writing – original draft, Visualization, Methodology, Investigation, Formal analysis, Data curation, Conceptualization. **Julie Davis:** Writing – review & editing, Validation, Project administration, Data curation. **James H. Stark:** Writing – review & editing, Supervision, Project administration, Funding acquisition. **Patrick H. Kelly:** Writing – review & editing, Writing – original draft, Visualization, Methodology, Investigation, Funding acquisition, Data curation.

## Consent to participate

Not applicable.

## Ethics approval

Not applicable.

## Code availability

Not applicable.

## Declaration of competing interest

The authors declare the following financial interests/personal relationships which may be considered as potential competing interests:

Patrick H. Kelly, Julie Davis, James H. Stark, and Agustín Estrada-Peña report financial support was provided by 10.13039/100004319Pfizer. Patrick H. Kelly, Julie Davis, and James H. Stark report a relationship with Pfizer that includes: employment and equity or stocks. Agustín Estrada-Peña reports a paid consultancy for Pfizer in connection with the development of this manuscript. The authors declare that this analysis was supported and jointly funded by Valneva and 10.13039/100004319Pfizer as part of their co-development of a Lyme Disease vaccine.

## Data Availability

All the raw data used for this study are available as Supplemental Material.

## Appendix A Supplementary data

Supplemental File S1. List of species occurrence downloads from Global Biodiversity Information Facility (GBIF) for modelling. Vertebrates: 1. GBIF.org (19 October 2024) GBIF Occurrence Download https://doi.org/10.15468/dl.es9vga 2. GBIF.org (19 October 2024) GBIF Occurrence Download https://doi.org/10.15468/dl.qfms85 3. GBIF.org (19 October 2024) GBIF Occurrence Download https://doi.org/10.15468/dl.mrpdsx 4. GBIF.org (19 October 2024) GBIF Occurrence Download https://doi.org/10.15468/dl.wurvz7 5. GBIF.org (19 October 2024) GBIF Occurrence Download https://doi.org/10.15468/dl.a94rdk Ixodes ticks: 1. GBIF.org (15 September 2024) GBIF Occurrence Download https://doi.org/10.15468/dl.h6b2tf Image 1

Supplemental File S2. Data on the performance of models for each genus of vertebrates included in this study, reported as hosts of *Ixodes ricinus* and/or competent reservoirs of *Borrelia burgdorferi* s.l. Threshold indicates the optimal value to convert the habitat suitability map into a binary presence/absence map (higher value is a better model). Other indexes include the area under the curve (AUC), sensitivity and specificity (false negatives and false positives), the proportion of correctly classified points. The kappa statistic is a method to evaluate the performance of models predicting presence/absence probability. Image 2

Supplemental File S3. A spatial datafile of the infection rates of *Borrelia burgdorferi in* host-seeking *Ixodes ricinus* nymphs collected in Europe. The dataset includes all the variables tested suitable for modelling (this study) including climate, actual records and modeled distribution of the vector tick, actual and modeled prevalence rates in ticks, and the modeled distribution of *Borrelia burgdorferi* s.l. The dataset is a GeoPackage that can be viewed with standard and open geostatistical software. Image 3
