# Supplementary material for: The niche of Borrelia burgdorferi sensu lato in Europe is predictable and mappable
Source: One Health. 2025 Dec 24;22:101313. doi: 10.1016/j.onehlt.2025.101313 (PMC12811546; doi:10.1016/j.onehlt.2025.101313)
Supplement: Supplementary file 1 — Supplemental File S1. List of species occurrence downloads from Global Biodiversity Information Facility (GBIF) for modelling. Vertebrates: 1. GBIF.org (19 October 2024) GBIF Occurrence Download https://doi.org/10.15468/dl.es9vga 2. GBIF.org (19 October 2024) GBIF Occurrence Download https://doi.org/10.15468/dl.qfms85 3. GBIF.org (19 October 2024) GBIF Occurrence Download https://doi.org/10.15468/dl.mrpdsx 4. GBIF.org (19 October 2024) GBIF Occurrence Download https://doi.org/10.15468/dl.wurvz7 5. GBIF.org (19 October 2024) GBIF Occurrence Download https://doi.org/10.15468/dl.a94rdk Ixodes ticks: 1. GBIF.org (15 September 2024) GBIF Occurrence Download https://doi.org/10.15468/dl.h6b2tf [file mmc1.docx]

**Supplemental File S1**. List of species occurrence downloads from Global Biodiversity Information Facility (GBIF) for modeling.

*Vertebrates:*

1. GBIF.org (19 October 2024) GBIF Occurrence Download <https://doi.org/10.15468/dl.es9vga>
2. GBIF.org (19 October 2024) GBIF Occurrence Download <https://doi.org/10.15468/dl.qfms85>
3. GBIF.org (19 October 2024) GBIF Occurrence Download <https://doi.org/10.15468/dl.mrpdsx>
4. GBIF.org (19 October 2024) GBIF Occurrence Download <https://doi.org/10.15468/dl.wurvz7>
5. GBIF.org (19 October 2024) GBIF Occurrence Download <https://doi.org/10.15468/dl.a94rdk>

*Ixodes ticks:*

1. GBIF.org (15 September 2024) GBIF Occurrence Download <https://doi.org/10.15468/dl.h6b2tf>
